# Study on the Properties of Basalt Fiber-Calcined Gangue-Silty Clay Foam Concrete for Filling Undermined Goaf Areas of Highways

**DOI:** 10.3390/ma18010047

**Published:** 2024-12-26

**Authors:** Yucong Yin, Qinglin Li, Yangpeng Zhang, Xiaodong Jiao, Pengrui Feng, Hexiang Zhang

**Affiliations:** 1 Guangxi Transportation Science and Technology Group Co., Ltd., Nanning 530007, China; 15700971929@163.com (Y.Y.); jxd991164424@163.com (X.J.); 2 Guangxi Key Lab of Road Structure and Materials, Nanning 530007, China; 3 College of Water Resources and Construction Engineering, Shihezi University, Shihezi 832000, China; drfxj666@163.com (P.F.); 13364984847@163.com (H.Z.); 4 Key Laboratory of Cold and Arid Regions Eco-Hydraulic Engineering of Xinjiang Production & Construction Corps, Shihezi 832000, China

**Keywords:** basalt fiber, calcined gangue, silty clay, mechanical properties, cubic compressive strength stress–strain curve, goaf-filled foam concrete

## Abstract

The collapse of surface goaf beneath highways can result in instability and damage to roadbeds. However, filling the goaf areas with foam concrete can significantly enhance the stability of the roadbeds while considerably reducing the costs of filling materials. This study analyzes the effects on destructive characteristics, mechanical properties, stress–strain curve features, and relevant metrics, while also observing the microstructure of basalt fiber-calcined gangue-silty clay foam concrete (BF-CCG-SCFC). The results indicate that the water–binder ratio significantly influences the cubic compressive strength, split tensile strength, and fluidity of BF-CCG-SCFC. Silty clay reduces the cubic compressive strength, split tensile strength, and fluidity of BF-CCG-SCFC. Conversely, an appropriate amount of calcined gangue and basalt fiber significantly increases the cubic compressive strength and split tensile strength, while decreasing fluidity. To satisfy the strength and fluidity requirements of the filler material in hollow areas, the optimal water–binder ratio for BF-CCG-SCFC is 0.55, the ideal mixing ratio of calcined gangue to silty clay is 2:2, and the basalt fiber content should be 1%. The study examines the influence of varying water–binder ratios, the combined proportions of calcined gangue and silty clay, and different basalt fiber contents on the elastic modulus, peak stress, and peak strain of BF-CCG-SCFC. Additionally, the water–binder ratio influences the matrix strength through the non-hydration reactions of doped particles, while gangue and clay induce a “gradient hydration effect” during the hydration process. The incorporation of basalt fibers enhances the mechanical interlocking between the fibers and the matrix.

## 1. Introduction

With the implementation of Chinese strategies such as “Western Development” and the rapid progress of urban construction, numerous highways, railways, and other transportation infrastructures must traverse goaf areas, facing risks of roadbed instability and damage due to potential collapses. Grouting and filling are essential methods for managing goaf areas, and foam concrete is widely used both domestically and internationally to fill large cavities [1,2,3,4]. Foam concrete effectively reduces the filling load, minimizes additional stress on the foundation, and inhibits movement and deformation of the overlying rock layer due to its lightweight, cost-effective, and adjustable properties [5,6,7,8], making it highly suitable as a filling material in goaf areas. Furthermore, in the context of the national strategy of “carbon peak, carbon neutral”, the research and development of green solid waste and resource-efficient materials have entered a new phase of opportunity. In traditional cement-bonded concrete filling processes, the cement content can account for 30% to 60% of the total filling material cost. Conversely, foam concrete plays a crucial role in solid waste management by significantly lowering the cost of filling materials and facilitating the efficient, green treatment of solid waste [9].

Recent global research on the mechanical properties of foam concrete has focused primarily on the impacts of various admixtures, including traditional mineral admixtures and novel fibers. Coal gangue, the primary industrial solid waste produced from coal mining in China [10], has garnered significant attention as a replacement for cementitious materials. Zhang Xin et al. [11] studied the mechanical properties and microstructure of gangue foam concrete, highlighting that the gangue content is crucial in influencing porosity, and a lower content can enhance mechanical properties. When rich in clay and calcareous minerals, thermal activation can enhance coal gangue’s properties and pozzolanic activity [12,13,14,15]. For instance, Dong Zuochao et al. [16] found that the activity of calcined gangue fine aggregate increased, which enhanced the compressive and flexural strengths of cement mortar.

The combination of cement and clay can complement each other’s deficiencies, forming a slurry with improved performance [17]. Since foam concrete has relatively low strength requirements, replacing cement with clay can significantly reduce grouting costs. Zhang et al. [18,19] investigated the compressive and flexural strengths of silt-based foam concrete, showing that it meets subgrade filling requirements while reducing cement use and costs. Du et al. [20] found that increasing clay content can significantly enhance the strength properties of foam concrete. Zhang Hongbo et al. [21] indicated that the compressive strength, flexural strength, and modulus of elasticity of pulverized soil-based foam concrete gradually decrease as the pulverized soil content and plasticity index increase. Current research primarily focuses on the effects of incorporating coal gangue or clay individually on foam concrete properties, whereas studies on the combined incorporation of calcined coal gangue and silty clay are limited.

Additionally, numerous scholars have noted that basalt fibers, with a density similar to that of concrete, exhibit excellent composition and compatibility [22,23,24], significantly enhancing the performance of foam concrete. Gencela Osman et al. [25] utilized basalt fibers compounded with silica fume to create highly durable foam concrete, achieving maximum compressive and splitting tensile strengths of 1.46 MPa and 3.07 MPa, respectively. The integration of basalt fibers was found to enhance the mechanical properties of the foam concrete. Shi Xinxin et al. [26] explored the pore structure of foam concrete modified with dispersible latex powder and basalt fibers. They determined that the compressive strength of foam concrete incorporating both dispersible latex powder and basalt fibers was significantly higher than that achieved with only basalt fibers. Wang Xinquan et al. [27] experimented with different proportions of basalt fibers and sisal fibers to develop a blended natural fiber roadbed foam concrete. The study indicated that when the volume fraction of blended fibers was adjusted to 0.3%, the foam concrete achieved optimal mechanical properties and durability.

This study utilizes basalt fibers as a reinforcement material, incorporating calcined coal gangue and silty clay into foam concrete to prepare basalt fiber-calcined coal gangue-silty clay foam concrete (BF-CCG-SCFC). The main factors are the water–binder ratio, the blending ratio of coal gangue and clay, and the content of basalt fiber. According to relevant standards, the workability of the foam concrete is primarily evaluated by flowability, compressive strength, and split tensile strength; therefore, the material undergoes cubic compressive tests, split tensile tests, and flowability tests to investigate failure modes, mechanical properties, stress–strain curves, and micro-mechanisms. The aim is to develop an economical novel foam concrete filler that meets the technical requirements for filling aged goaf areas, effectively reducing the costs of goaf management. This study fully utilizes the industrial solid waste gangue widely distributed in the coal mining area as well as the powdery clay, and this experimental study is able to prove that BF-CCG-CLLCC meets the working performance (strength and fluidity) of the air-mining area, injects innovative vitality into the traditional concrete technology, and provides an important support for the development of new types of air-mining area filling materials. The research findings possess significant experimental reference value and practical implications for the application of foam concrete in engineering, contributing to the enhancement of road traffic safety in mining areas.

## 2. Materials and Methods

### 2.1. Raw Materials

Basalt fiber-calcined coal gangue-silty clay foam concrete (BF-CCG-SCFC) consists of cement, calcined coal gangue, silty clay, basalt fibers, polymer composite cement foaming agent, and water.

Cement: P.O 42.5 ordinary Portland cement produced by Tianneng Cement Co., Ltd. (Shihezi, China) was used; its basic properties and chemical composition are listed in Table 1 and Table 2, respectively.

**Figure 1:**
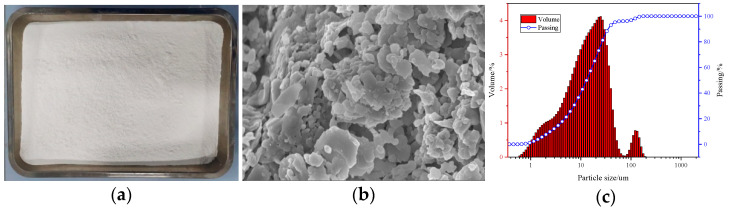
Calcined gangue: (**a**) macro-plot; (**b**) SEM; (**c**) particle size distribution.

Calcined coal gangue is shown in Figure 1; coal gangue was sourced from an industrial park in northern Xinjiang.

The block gangue is initially crushed, then passed through a 5 mm sieve, followed by calcination at 750 °C for 2 h in a muffle furnace (Figure 1). After cooling, it is further crushed to obtain activated white gangue powder with a particle size of less than 45 μm. The chemical composition is presented in Table 3.

**Figure 2:**
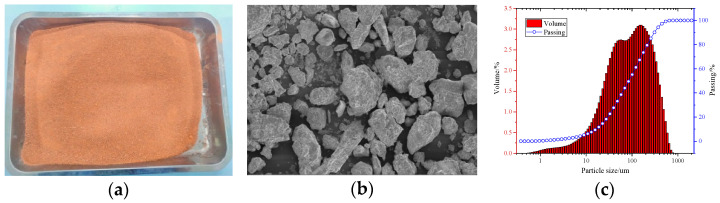
Silty clay: (**a**) macro-plot; (**b**) SEM; (**c**) particle size distribution.

Silty clay (Figure 2) was sourced from the slopes of mountainous regions in Xinjiang, the silty clay is purified by passing through a 1 mm standard sieve and then dried in an oven at 60 °C until a constant weight is achieved. The silty clay particles exhibit a well-graded distribution.

Polymer composite cement foaming agent: Produced by Shandong Province Weihai Zhongsheng New Building Materials Co., Ltd. (Weihai, China). The performance tests of the foaming agent comply with the ‘Technical specification for foamed mixture lightweight soil filling engineering’ [28]. When diluted to 60 times, the foaming agent exhibits minimal water secretion and the lowest settlement distance. The physical properties of the indexes are detailed in Table 4.

**Figure 3:**
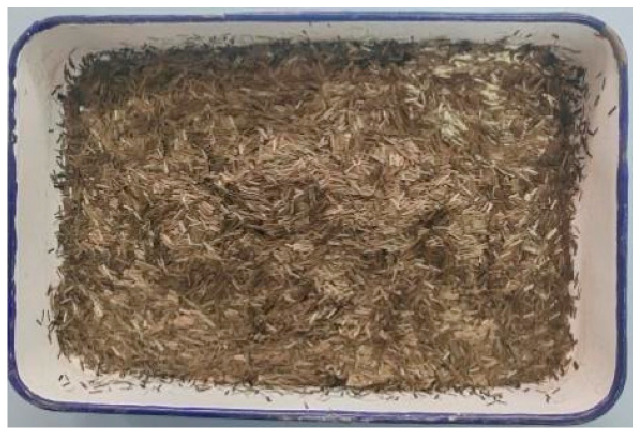
Basalt fiber (water: tap water intended for daily consumption).

Basalt fiber is shown in Figure 3; short-cut basalt fibers were provided by Xinjiang Woyu New Material Co. (Urumqi, China).

Testing adheres to standard ‘Basalt fiber and product for highway engineering’ [29]. The primary performance parameters are outlined in Table 5.

### 2.2. Specimen Preparation

**Figure 4:**
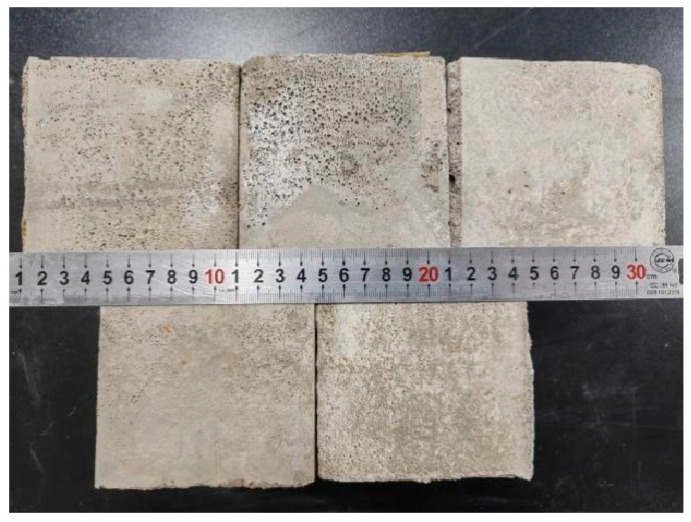
Basalt fiber calcined coal gangue silty clay foam concrete.

This study utilizes the prefabricated foam method to prepare foam concrete (Figure 4). The primary preparation steps are as follows:

1. The composite blowing agent is diluted with water at a 1:60 ratio to form a solution. This solution is uniformly stirred and subjected to high-speed mixing using a micro-cement foaming machine to achieve physical foaming and create foam clusters.
2. Mixing the Components: Based on the target wet density and mixture proportions, the required mass of cement for each batch is calculated using a mass method. The masses of calcined gangue, silty clay, basalt fiber, and water are then precisely weighed. The dry materials are mixed in a mixer for 150 s to ensure thorough integration. Afterward, the specified quantity of water is added, and the mixture is blended for an additional 3 min.
3. Incorporating the Foam: A specific amount of foam is weighed and mixed with the cement slurry for 3 min. Once the slurry is homogeneously mixed with no surface bubbles, a fluidity test is promptly conducted.
4. The mixture is then poured into a 100 mm square mold and gently vibrated manually to compact it. The surface is covered with cling film. After 24 h of resting, the mold surface is leveled, and the sample is de-molded using an air gun and labeled. The samples are then stored in a standard curing room at a temperature of (20 ± 2) °C and relative humidity exceeding 95%, curing them to specific ages (3, 7, and 28 days). Three parallel tests are conducted for each group’s mobility test, and three 100 mm × 100 mm × 100 mm cube specimens are prepared for compression and split tensile tests at each specified age. The results are then averaged.

### 2.3. Test Apparatus and Method

The evaluation of the working performance of foam concrete specimens is conducted sequentially using the following methods:

1. Fluidity: The flow value of the freshly mixed slurry is determined using the cylinder method outlined in the Technical specification for application of foamed concrete [30]. A hollow cylinder with a height and inner diameter of 80 mm is utilized for this measurement.
2. Cubic Compressive Strength and Splitting Tensile Strength: Tests are performed on specimens sized 100 mm × 100 mm × 100 mm, in accordance with the specifications in the Technical specification for application of foamed concrete [30]. Since non-standard specimens are used, compressive strength values are adjusted with a size conversion factor of 0.95, and splitting tensile strength values are adjusted using a factor of 0.85.
3. Elastic Modulus: The secant modulus is calculated between the origin and the point corresponding to 0.4 *f_c_* on the ascending segment of the stress–strain curve; the average value is derived from three specimens for each group.

The secant modulus (*E_s_*): The secant modulus is calculated based on two points on the curve; usually 0.4 f_c_ (0.4 times the peak stress) is chosen as the reference point, and the equation is as follows:

$$E_{s}=\frac{\sigma_{1}-\sigma_{0}}{\varepsilon_{1}-\varepsilon_{2}}$$

*σ*_1_ = 0.4 *f_c_*: The reference point stress.

*σ*_0_ = 0: The initial stress.

*ε*_1_: The strain value corresponding to *σ*_1_.

*ε*_0_ = 0: The initial strain.

4. Peak Stress and Peak Strain: The peak stress (*σ_p_*) represents the compressive strength of the foam concrete, while the corresponding deformation is identified as the peak strain (*ε_p_*).
5. Scanning Electron Microscopy (SEM) Test: Small pieces (<1 cm^3^) from the midsection of specimens exhibiting cubic compressive and splitting tensile damage are soaked in anhydrous ethanol to halt hydration. These samples are then dried in a vacuum oven at (60 ± 5) °C, flattened with sandpaper, and coated with gold for imaging using a Zeiss 360 sigma SEM (Oberkochen, Germany).

### 2.4. Single-Factor Test Design

Based on the Technical specification for application of foamed concrete [30], a single-factor proportionality test was conducted to design foam concrete filler material with a target dry density of 800 kg/m^3^. The study investigated the influence of the water–binder ratio, the proportion of calcined gangue and silty clay, and the quantity of basalt fiber admixture, with each factor tested at five distinct levels. A controlled variable approach was employed to assess the impact of these factors on the physical and mechanical properties, including cubic compressive strength, split tensile strength, and flowability.

Following preliminary mix design experiments, the water–binder ratio for foam concrete was set between 0.4 and 0.6. Calcined gangue and silty clay were combined in varying proportions to replace 40% of the cement by mass. The ratios of m(calcined gangue) and m(silty clay) were 4:0, 3:1, 2:2, 1:3, and 0:4, while maintaining constant proportions of the other raw materials. Basalt fibers were introduced as reinforcement. The experimental plan includes 15 mix proportions, as detailed in Table 6 and Table 7.

## 3. Results

To investigate the fracture characteristics, mechanical properties, and flow properties of BF-CCG-SCFC specimens, a comprehensive evaluation of cubic compressive strength, splitting tensile strength, and flowability was conducted. The study examines the effects of the water–binder ratio, the combined proportions of calcined gangue and silty clay, and the content of basalt fibers on various performance metrics. Additionally, the stress–strain curve patterns of the compressive strength of the cubes and the stress–strain characteristic indexes, such as the modulus of elasticity and peak strain, were analyzed, and finally, the microscopic mechanism was investigated.

### 3.1. Destruction Process and Characteristics

#### 3.1.1. Cubic Compressive Failure Process and Characteristics

**Figure 5:**
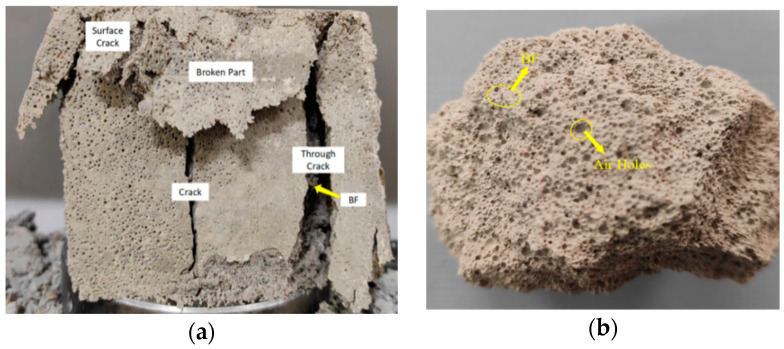
BF-CCG-SCFC cubic compression test damage pattern: (**a**) surface damage section; (**b**) internal damage section.

The typical failure modes of BF-CCG-SCFC cubic compressive specimens are illustrated in Figure 5.

Numerous pores with an average diameter of approximately 1 mm are distributed throughout the specimens. The damage progression is characterized by distinct phases: linear elastic deformation, crack initiation, crack development, and eventual failure.

In the initial loading stage, the cracks in the foam concrete specimens remained largely undeveloped, with no apparent surface deformation. As loading continued, micro-cracks began to manifest, compromising the contact surfaces between the specimens and the indenter. Concurrently, small pores gradually ruptured, leading to epidermal peeling and producing slight crushing sounds as surface cracks developed and vertical fissures emerged. As the load increased, the specimens exhibited plastic deformation, with noticeable compression in the upper sections. Cracks progressively widened, new fractures formed, and fragments began to detach from the specimens. Ultimately, nearly half of the specimen was crushed, with cracks propagating from the upper end downward; the cracks are not simply downward, but also penetrate internally, gradually forming an angle with the vertical. Observation of the crack development and damage pattern of each specimen showed that with the increase in FC fiber doping, the initial direction of the cracks changed from longitudinal cleavage damage to diagonal cross-section shear damage. The damage cracks in the unreinforced foam concrete were vertically oriented, while the cracks in the foam concrete with basalt fibers added gradually formed an angle with the vertical direction.

The damage primarily consisted of small cracks, with fewer extending through the entire specimen. Upon failure, the basalt fibers at the crack termini acted as bridges, facilitating stress transfer and effectively mitigating further crack propagation, thereby enhancing the compressive strength of the specimen.

#### 3.1.2. Splitting Tensile Failure Process and Characteristics

**Figure 6:**
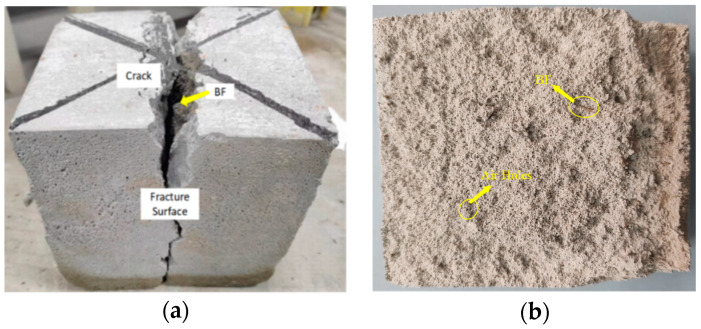
BF-CCG-SCFC split tensile test damage pattern: (**a**) surface damage cross-section; (**b**) internal damage section.

Figure 6 illustrates the splitting tensile failure mode of the specimen. In the initial loading stage, the specimen remains in an elastic phase. As the load increases, cracks first appear at the spacers at both ends of the specimen and gradually expand toward the center. With further load application, the cracks along the splitting plane extend from both ends until they penetrate the entire specimen, accompanied by slight cracking sounds, ultimately resulting in splitting failure. Upon reaching the failure load, the specimen splits directly into two halves, with the failure cracks oriented vertically and aligned with the direction of the load application. The fracture surface retains good integrity, presenting an “I” shape that closely resembles the ideal splitting model of foam concrete. The cracks on the fracture surface can be categorized into primary and secondary cracks. The specimen remains largely intact owing to the bridging and binding effect of basalt fibers. The surface predominantly exhibits one large, long crack accompanied by several smaller, shorter cracks. Upon closer inspection, fibers can still be seen connecting within the specimen.

### 3.2. Influence of Factors on the Basic Properties of BF-CCG-SCFC

The water–binder ratio, the proportion of calcined gangue and silty clay re-mixing, and the basalt fiber content are critical factors influencing the mechanical properties and fluidity of foam concrete. To investigate the effects of these variables on the compressive strength, splitting tensile strength, and fluidity of foam concrete cubes, specimens were prepared and cured for 3, 7, and 28 days. The results of their mechanical properties and fluidity performance are summarized as follows.

#### 3.2.1. Water–Binder Ratio

**Figure 7:**
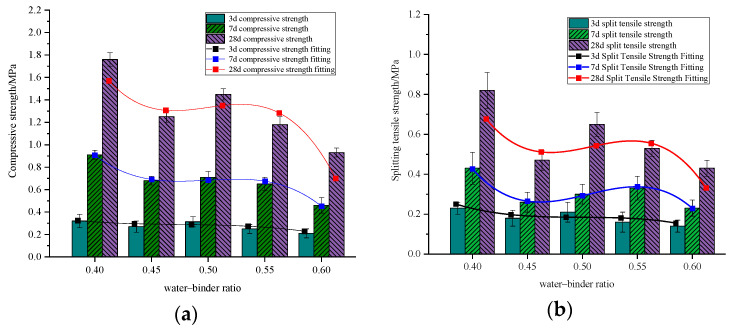
Effect of water–binder ratio on mechanical properties of BF-CCG-SCFC: (**a**) cubic compressive strength; (**b**) splitting tensile strength.

As shown in Figure 7, when the water–binder ratio increases, the trends in both compressive strength and splitting tensile strength of specimens cured for different ages exhibit that cubic compressive strength and split tensile strength of specimens maintained at different ages oscillate in the same manner as the water-cement ratio increases. When the water–binder ratio is 0.4, both compressive and splitting tensile strengths reach their maximum values, with a second peak at a ratio of 0.5. However, when the water–binder ratio exceeds 0.5, both strengths significantly decrease, averaging a reduction of 19.8% for every 0.05 increase in the ratio, reaching their minimum values at a ratio of 0.6. This behavior can be attributed to the following: when the water–binder ratio is too low, there is insufficient water for cement hydration, leading to water absorption from the foam, which can cause foam rupture and result in fewer internal pores, thereby increasing specimen strength. Conversely, a high water–binder ratio leads to excessive interconnected porosity during cement hardening, increasing friction between the foam and the slurry and reducing slurry consistency. This results in more foam rupture, uneven foam distribution, and increased internal defects, ultimately reducing specimen strength. At a water–binder ratio of 0.5, cement hydration is adequate, the foam is evenly distributed, and porosity is moderate, resulting in optimal performance and higher specimen strength. According to the Technical Specification for Design and Construction of Cast-in-situ Foamed Lightweight Soil Subgrade [31], for high-grade highways, the 28-day unconfined compressive strength of foam concrete should not be less than 0.6 MPa when the top surface of the foam concrete base is more than 0.8 m from the road surface. Since the compressive strength of the specimens exceeds this requirement, the foam–concrete mix ratio meets the strength specifications for the roadbed.

**Figure 8:**
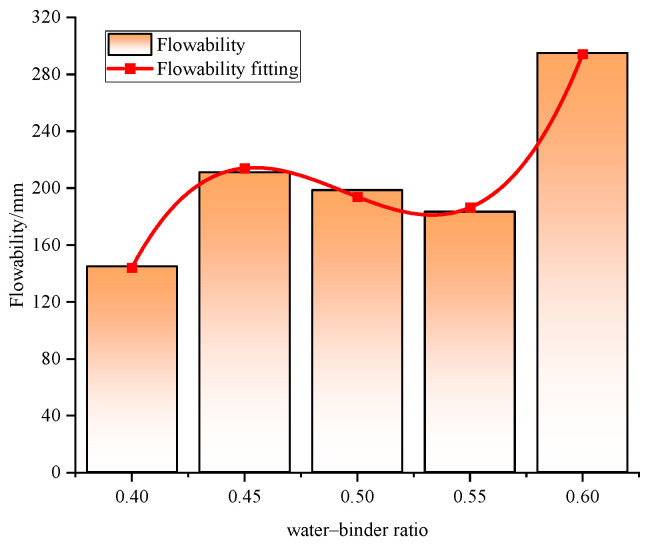
Effect of water–binder ratio on the flowability of BF-CCG-SCFC.

The water–binder ratio also significantly influences the flowability of foam concrete slurry (Figure 8). Generally, flowability improves as the water–binder ratio increases, showing a positive correlation, with flow values ranging from 144.8 to 295 mm. As the proportion of solids increases within the water–binder ratio, the flow value decreases, indicating poorer flowability. This suggests that reducing water content notably impacts flowability. At a constant admixture dosage, larger water–binder ratios yield higher flow values compared to smaller ratios, indicating that increased water content or reduced cement paste enhances flowability. In high-grade highway engineering, foam concrete should have a flow value between 160 and 200 mm. Therefore, a water–binder ratio of 0.5 is optimal, as it achieves the necessary flow value while the 28-day compressive strength satisfies both mobility and strength requirements for the material.

#### 3.2.2. Proportion of Calcined Gangue and Silty Clay Re-Mixing

**Figure 9:**
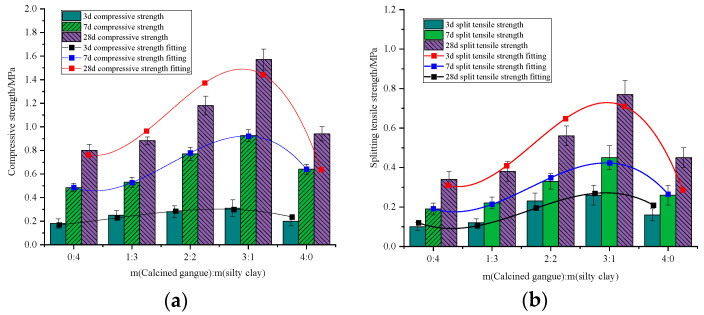
Effect of calcined gangue and silty clay compounding ratio on mechanical properties of BF-CCG-SCFC: (**a**) cubic compressive strength; (**b**) splitting tensile strength.

The mixing ratio of calcined gangue to silty clay ranges from 0:4 to 4:0, and cubic compressive strength and split tensile strength of specimens maintained at different ages oscillate in the same manner as the water-cement ratio increases (Figure 9). Initially, strength increases with the amount of admixture before decreasing, with calcined gangue demonstrating a more pronounced effect on strength enhancement. Foam concrete with specific proportions of calcined gangue and silty clay exhibits higher strength than mixtures containing only calcined gangue or silty clay. When only clay is used, the compressive strength of foam concrete is at its lowest, indicating that clay’s contribution is limited and can even be detrimental. A higher proportion of calcined gangue results in increased compressive strength, highlighting the significant positive impact of calcined gangue on strength enhancement. Mixing calcined gangue and silty clay yields better results, particularly at the optimal ratio of 3:1, which achieves peak strength; the cubic compressive strength and split tensile strength of 28d specimens are 50.6% and 54.5% higher than those of clay alone. This phenomenon can be attributed to calcined gangue, which not only exhibits pozzolanic effects but also contributes to the structural framework and compensates for pore defects. Increasing the proportion of calcined gangue reduces the occurrence of cracks and large pores, while the bond-filling action of C-S-H and C-A-H gels enhances the integrity and pore structure of foam concrete, resulting in smaller pores, increased densification, and higher compressive strength. Improved pore size leads to denser foam concrete, thereby enhancing compressive strength. However, excessive gangue can create weak surfaces, negatively impacting both compressive and tensile strength.

**Figure 10:**
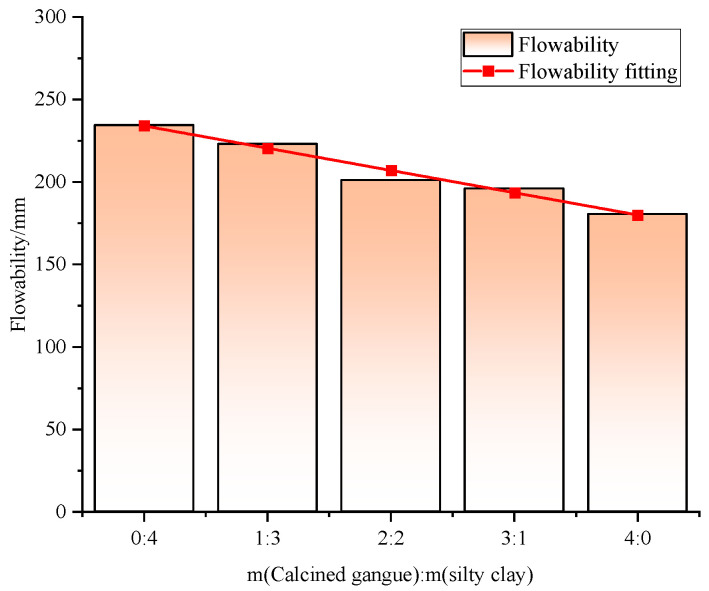
Effect of gangue and clay compounding ratio on the flowability of BF-CCG-SCFC.

Results indicate that the flowability of foam concrete tends to decrease linearly as the calcined gangue dosage increases and the silty clay dosage decreases (Figure 10). This occurs because excessive gangue and insufficient silty clay lead to the formation of weak surfaces, adversely affecting flowability. Considering the fluidity requirements for filling materials in goaf areas (160 mm to 200 mm), optimal fluidity is achieved at gangue–clay ratios of 3:1 and 4:0. Therefore, to ensure both strength and mobility, the gangue–clay mixing ratio should be maintained at 3:1.

#### 3.2.3. Basalt Fiber Dosage

**Figure 11:**
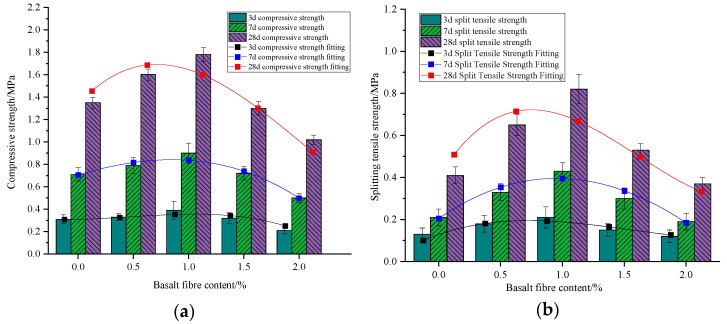
Effect of basalt fiber doping on mechanical properties of BF-CCG-SCFC: (**a**) cubic compressive strength; (**b**) splitting tensile strength.

It is evident that as the dosage of basalt fiber gradually increases, both the compressive strength and splitting tensile strength of foam concrete initially rise and then decline (Figure 11). At a basalt fiber dosage of 1%, both strengths peak. When the basalt fiber dosage is below 1%, increases correlate with higher compressive strength, averaging a 25.2% increase per 0.5% dosage increment, and an increase in split tensile strength by 37.8%. However, beyond 1%, further increases result in a decrease in strength. Relative to the baseline group (0% basalt fiber dosage), compressive strength can decrease by up to 29.6% at a 1% dosage, and split tensile strength can decrease by up to 34.9% at a 1% dosage; at 2%, the compressive strength reduces to 73% of the baseline, and the split tensile strength reduces to 87.8% of the baseline. Mechanistic analysis indicates that with less than 1% fiber, the uniformly distributed fibers restrict transverse expansion under pressure, inducing a crack-blocking effect that controls crack propagation and enhances compressive strength. Beyond a 1% dosage, fibers become prone to bending under pressure, and increased fiber content leads to more internal pores and fiber agglomeration, resulting in matrix defects and a subsequent reduction in strength.

**Figure 12:**
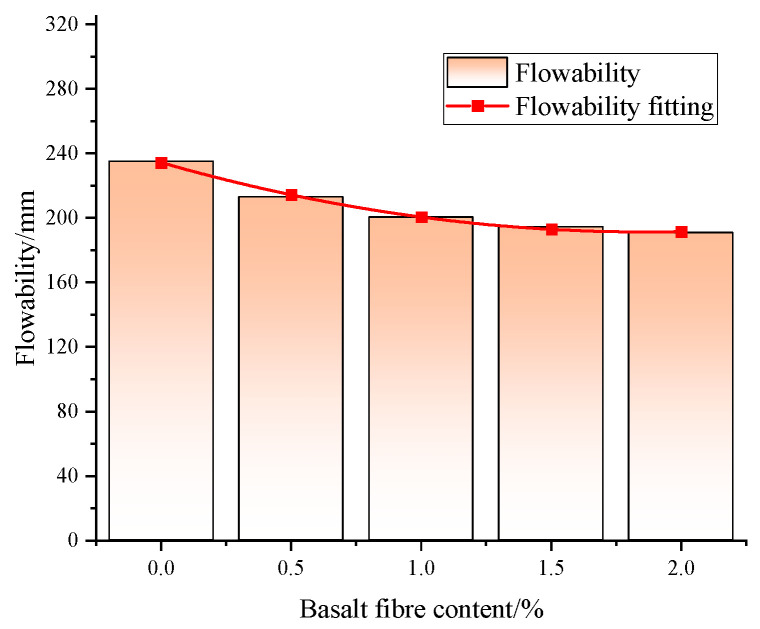
Effect of basalt fiber doping on the flowability of BF-CCG-SCFC.

Overall, incorporating fiber reduces the flowability of foam concrete mixes, with this reduction gradually lessening at higher basalt fiber dosages (Figure 12). Compared to the baseline group (0% basalt fiber dosage), flowability decreases by 10.21%, 14.68%, 17.32%, and 18.81% at basalt fiber dosages of 0.5%, 1%, 1.5%, and 2%, respectively. Flowability values range from 190.8 to 235.2 mm, indicating a significant effect of basalt fiber dosage on flowability. Mechanistic analysis reveals that the random distribution of fibers within the matrix forms a spatial network akin to a ‘skeleton’ enhancing frictional resistance among fibers and between fibers and the matrix, thereby reducing matrix fluidity. Despite being hydrophobic, basalt fiber still adsorbs some water upon incorporation into the matrix, further reducing fluidity. The agglomeration caused by increased basalt fiber dosage diminishes the fiber’s water adsorption effect, thereby slowing the rate of flowability decline. To meet the mobility requirements for foam concrete in the hollow area filling, the basalt fiber dosage should be maintained at 1%, balancing mobility and compressive strength to ensure technical compliance for grouting applications.

As shown in Table 8, in order to meet the working performance requirements of strength and fluidity of filling materials in the hollow area, when considering the role of a single factor in each influencing factor, the optimal water-cement ratio of BF-CCG-SCFC is 0.5, and the ideal mixing ratio of calcined gangue and pulverized clay is 3:1, and the optimal content of basalt fibers should be 1%.After considering all the influencing factors, the optimal water cement ratio of BF-CCG-SCFC is 0.55, the ideal mixing ratio of calcined gangue and powdery clay is 2:2, and the optimal basalt fiber content should be 1%.

Gray correlation analysis was used to accurately describe the findings obtained in Section 3.2. Gray correlation analysis is to calculate the correlation degree between the characteristic sequence and the parent sequence, here to calculate the correlation between each index and the influencing factors. From the gray correlation results (Table 9), it can be seen that the gray correlation between the 28 d compressive strength of the specimen and each influencing factor is in the following order from large to small: water-cement ratio > basalt fiber doping > m(gangue):m(clay); the gray correlation between the 28d splitting tensile strength of the specimen and each influencing factor is in the following order from large to small: water-cement ratio > basalt fiber doping > m(gangue):m(clay). Therefore, the water-cement ratio is the most important factor affecting the strength of the specimens. In addition, the gray correlation between the 28d compressive strength of the specimen and each influencing factor is in the following order: water-cement ratio > m(gangue):m(clay) > basalt fiber doping. Therefore, the water-cement ratio is the most important factor affecting the fluidity of the specimens.

### 3.3. Stress–Strain Curve of Compression Resistance of BF-CCG-SCFC Cube

**Figure 13:**
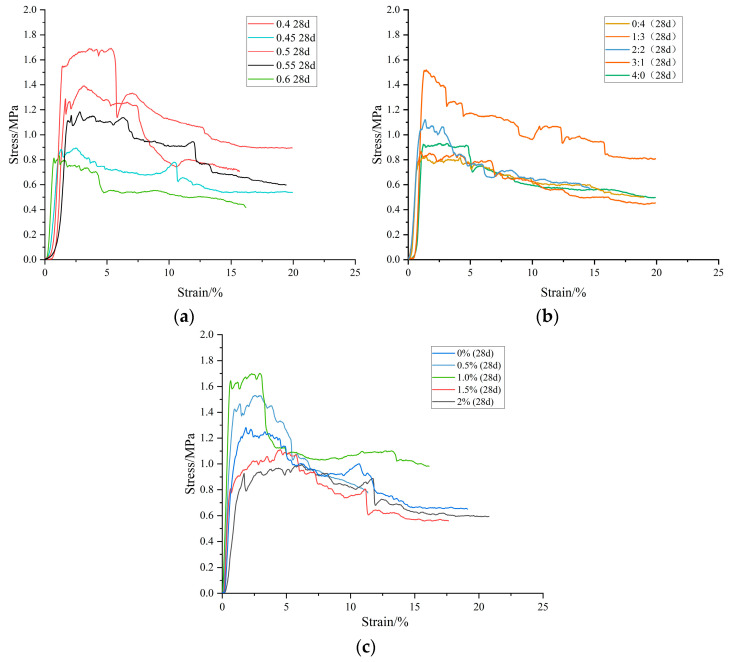
Stress–strain curves of BF-CCG-SCFC under different factors (28 d): (**a**) water–binder ratio; (**b**) calcined gangue and silty clay mixing ratio; (**c**) basalt fiber mixing amount.

The stress–strain curve serves as a vital tool for understanding the compression characteristics of foam concrete, revealing both elastic and plastic deformation stages. Analyzing this curve allows us to delve into the damage mechanisms that occur in foam concrete cubes under compression (Figure 13). In summary, the stress–strain curve of foam concrete narrates the journey of the material from compaction to deformation, offering insights into its unique behavior under stress. Understanding these stages is essential for optimizing foam concrete’s performance in various applications.

**Figure 14:**
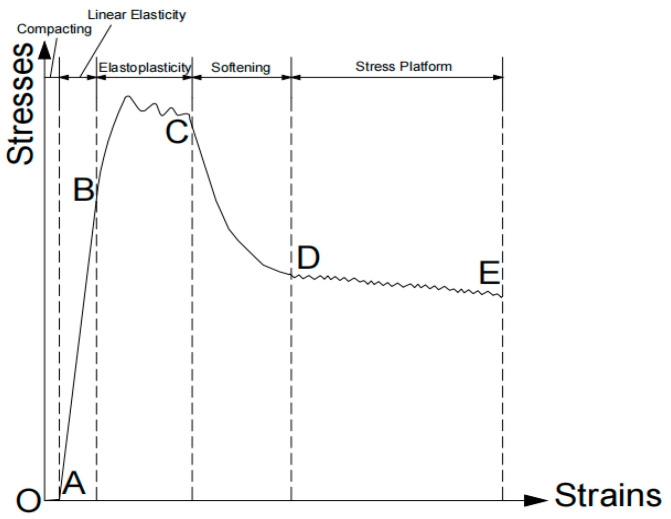
Cubic compressive stress–strain curve modelling of BF-CCG-SCFC.

The focus here is on foam concrete specimens cured for 28 days. The stress–strain curve of foam concrete specimens cured for 3 days and 7 days exhibits minor undulations, whereas specimens cured for 28 days show more pronounced undulations. The reason for this is that the relative slip between the fibers and the cement matrix is significantly reduced after longer curing times. This makes the single fiber contribute more to the strength of the specimen. As a result, when a single fiber breaks or pulls away from one of the ends, the stress drop is more pronounced than that observed at shorter curing times. As the curing age increases, the modulus of elasticity of foam concrete rises, while the peak strain decreases. The stress–strain curve changes consistently across different curing ages. Consequently, a uniform stress–strain curve model is applicable for describing all specimens (Figure 14).

The deformation and damage process of foam concrete typically progresses through five distinct stages (using the 28-day specimen as an example): compaction, linear–elastic, elastic–plastic, post-peak deformation, and stress platform stage.

1. Compaction Stage (OA section): At the outset, the specimen undergoes rapid compaction, closing pores and cracks under pressure. Stress gradually increases with strain, and during this phase, the internal voids from the foam remain largely intact, akin to compressing a sponge.
2. Linear Elasticity Stage (AB section): Here, the stress rises linearly with strain, resulting in a near-linear stress–strain curve that signifies elastic deformation. The specimen as a whole supports the external force, leading to a significant stress change with minimal slope variation. This is due to the foam concrete’s pore wall skeleton providing initial load-bearing capacity, similar to how a network of beams supports a structure.
3. Elastic–Plastic Stage (BC section): This stage marks the transition from elastic to elastic–plastic deformation, beginning at the specimen’s cracking point. As strain intensifies, the slope of the stress–strain curve diminishes, indicating that while stress approaches its peak, the strain grows at a faster rate. This creates a slightly convex curve. Unlike the elastic stage, microcrack expansion and new crack formation occur here, leading to a noticeable decrease in the modulus of elasticity.
4. Softening Stage (CD section): Once the stress surpasses its peak, the curve enters a descending phase, indicating damage to the specimen. Stress decreases gradually, forming an oblique linear segment where the curve flattens out. This behavior is attributed to the rotation and misalignment of internal particles, with plastic deformation becoming the dominant characteristic.
5. Stress Platform Stage (DE section): After reaching maximum stress, a residual stress level is sustained over a significant strain range, creating a pronounced stress platform. The presence of millimeter-sized closed vapor bubbles within the foam concrete contributes to this phenomenon, as these bubbles compress easily under pressure, promoting macroscopic plastic deformation. Despite the stress reduction, the overall cementitious structure remains intact, providing excellent deformation capacity and ductility—qualities that make foam concrete particularly suitable for applications like filling goaf areas.

### 3.4. Characteristic Indicators of Cubic Compressive Stress–Strain Curve

The key characteristic indices of the stress–strain curve include peak stress, peak strain, and elastic modulus. Peak stress is a critical indicator of the cubic compressive strength of foam concrete, and its variation patterns closely align with those of cubic compressive strength. Therefore, the trends discussed in Section 3.2 are applicable here and will not be reiterated.

#### 3.4.1. Modulus of Elasticity

**Figure 15:**
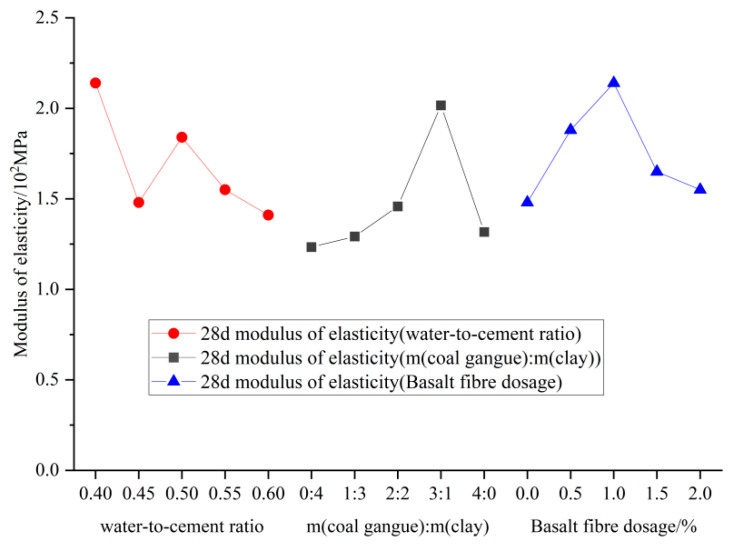
Effect of factors on the elastic modulus of BF-CCG-SCFC.

Figure 15 illustrates the variations in elastic modulus for each specimen of BF-CCG-SCFC.

The trend in elastic modulus closely mirrors that of peak stress. The modulus of elasticity first decreases with increasing water–binder ratio, followed by an increase, and then a decrease again, resulting in an overall downward trend. This behavior occurs because, at both excessively high and low water–binder ratios, the active SiO_2_ in metakaolin cannot adequately react with Ca(OH)_2_, the hydration product of cement, during the secondary hydration reaction. In contrast, at an optimal water–binder ratio, active SiO_2_ effectively engages with Ca(OH)_2_, promoting secondary hydration and producing additional C-S-H gel. This reaction generates more C-S-H gel, which fills micro-cracks and pores at the boundaries of new and old mortar within the matrix, thereby enhancing the material’s internal structure.

The modulus of elasticity of foam concrete increases with a higher proportion of calcined gangue, while a corresponding increase in silty clay content leads to a decrease. This trend shows an initial rise followed by a decline, with values ranging from 1.21 to 2.06 MPa. The proportion of gangue is the primary factor influencing the modulus of elasticity. This is primarily due to the moderate addition of calcined gangue, which enhances the compressive strength of foam concrete cubes, thereby increasing the modulus of elasticity. However, when the calcined gangue proportion exceeds optimal levels, it hinders the hydration reaction and reduces structural density, leading to decreased strength and modulus of elasticity.

The figure also indicates that the modulus of elasticity initially rises and then falls with the addition of basalt fiber; its value is comparatively strongly affected by fiber content. The maximum modulus was observed at a basalt fiber dosage of 1%, representing a 38.2% increase compared to specimens without basalt fiber. This increase is largely attributed to the enhancement of the aggregate-cementitious bond interface by basalt fiber, which creates additional bonding surfaces between the fibers and the matrix. This results in a more homogeneous and densely packed foam concrete. The modulus of elasticity decreased by 23.2% and 25.4% at basalt fiber dosages of 1.5% and 2%, respectively, compared to 1% basalt fiber dosage, indicating a significant decrease in the modulus of elasticity. Evidently, excessive fiber content can lead to fiber agglomeration, causing defects in the bond interface and a reduction in the modulus of elasticity.

#### 3.4.2. Peak Strain

The peak strain of BF-CCG-SCFC decreases with the increase in the water–binder ratio, which indicates that the water–binder ratio has a significant effect on the plastic deformation of this material, and the larger the water–binder ratio is, the peak strain decreases gradually, which indicates that the amount of plastic deformation is smaller when the water–binder ratio is larger, and the increase in the water–binder ratio accelerates the process of plastic deformation and shortens the amount of deformation in plastic deformation. The plastic deformation of this foam concrete is not obvious until the peak stress is reached. However, once the peak stress is exceeded, the deformability of the material increases. It is worth noting that the total deformation observed at specimen failure is much larger than that of plain concrete.

**Figure 16:**
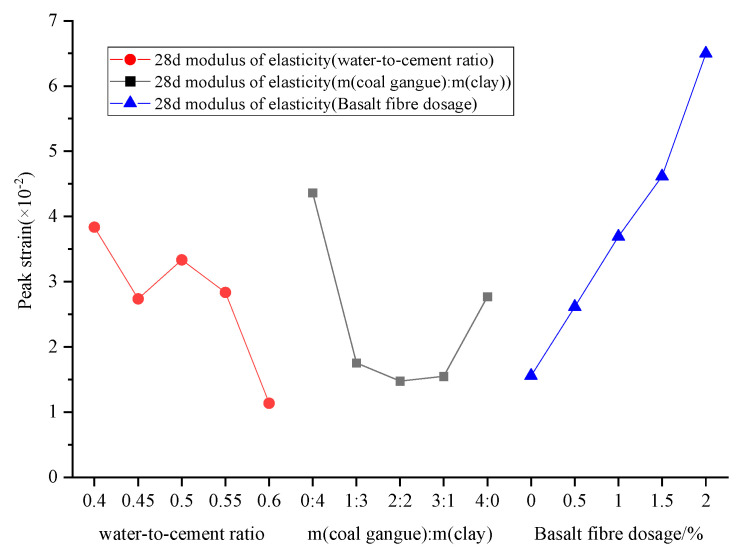
Relationship between factors and BF-CCG-SCFC peak strain.

There is an inverse correlation between peak strain and peak stress in BF-CCG-SCFC. When the calcined gangue content is below 30%, the peak strain significantly decreases compared to the benchmark foam concrete (Figure 16). This reduction is primarily attributed to the enhanced cubic compressive strength resulting from calcined gangue addition, which increases the brittleness of the foam concrete and subsequently lowers its peak strain. Conversely, when calcined gangue content exceeds 30%, the peak strain gradually increases. The reason is that the inherent strength of silty clay is lower than that of cement, the plasticity of silty clay is large, and an increase in silty clay substitution leads to a large rise in peak strain, indicating that silty clay has a more pronounced effect on peak strain than calcined gangue.

The incorporation of basalt fiber has a significant positive effect on peak strain, as the basalt fiber admixture increases, the peak strain gradually increases linearly. Compared to baseline (BF-0), peak strain increases by an average of 1.13 for every 0.5% increase in basalt fiber incorporation. This improvement can be attributed to the favorable tensile properties of basalt fiber. During the axial compression process, the transverse deformation within the matrix allows the fiber to restrain crack propagation, effectively altering the uniaxial longitudinal compression state and enhancing both the ductility and load-bearing capacity of the matrix.

### 3.5. Micro-Mechanism Analysis

**Figure 17:**
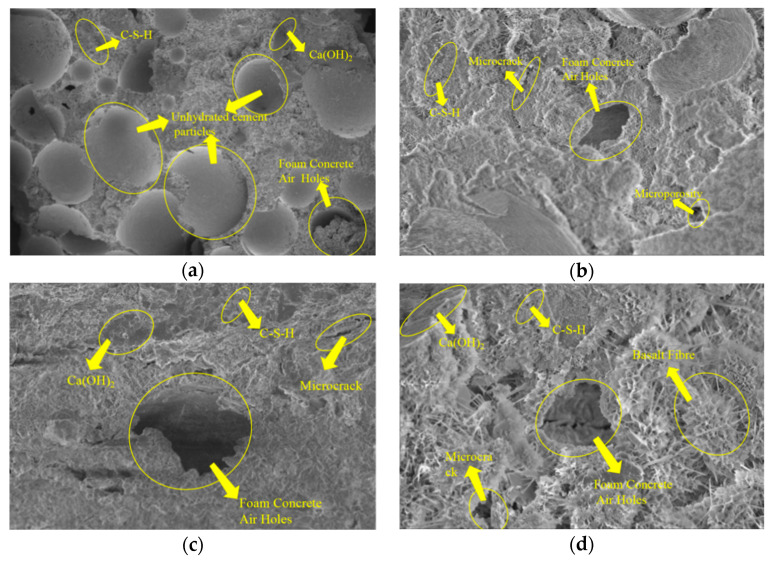

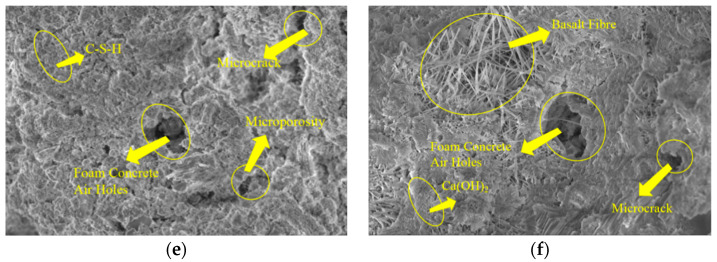
SEM image of BF-CCG-SCFC specimen: (**a**) water–binder ratio of 0.4; (**b**) water–binder ratio of 0.6; (**c**) m(calcined gangue):m(silty clay) = 4:0; (**d**) m(calcined gangue):m(silty clay) = 0:4; (**e**) fiber-free; (**f**) blended with basalt fibers.

To investigate the mechanisms by which BF-CCG-SCFC influences mechanical properties, scanning electron microscopy (SEM) was employed to examine the damage sections of BF-CCG-SCFC specimens. Figure 17 illustrates the microstructure of the specimen section following the cubic compression test; its analysis reveals the relationship between the microstructure and the macroscopic properties of the foam concrete specimens.

Figure 17a,b show that in foam concrete with a water–binder ratio of 0.4, spherical particles mainly consist of unhydrated admixture particles. Conversely, foam concrete with a water–binder ratio of 0.6 displays a scarcity of unhydrated cement particles, instead revealing a predominance of prismatic particles and a higher presence of C-S-H gels. The denser calcium alumina phase contributes to a more compact structure, indicating a more thorough hydration reaction. Upon magnification, there is a clear abundance of flocculent hydrated calcium silicate gels (C-S-H) and flaky calcium hydroxide crystals (Ca(OH)_2_), which fill the internal structure of the matrix and enhance overall strength.

Figure 17c,d highlight the inherent activity of calcined gangue and silty clay, along with their differing physical and chemical properties, leading to a “gradient hydration effect” during hydration. The varying material properties result in differences in hydration behavior. When calcined gangue is added excessively, it may not participate in secondary hydration; however, its small size allows it to fill capillary pores in the matrix, increasing structural density. This microstructural phenomenon explains the significant increase in compressive strength. An optimal amount of heat-activated calcined gangue enhances cement hydration, while excessive amounts may lead to increased water absorption and inhibit hydration. Additionally, the flaky structure of calcium hydroxide can be brittle, potentially diminishing the strength of the foam concrete.

Figure 17e shows that the foam concrete matrix without fibers has a monotonous and loosely structured appearance, with significant gaps at the aggregate-matrix interface. Noticeable hydration products, such as fibrous C-S-H and Ca(OH)_2_, are present within the matrix. Figure 17f presents SEM images of the BF-CCG-SCFC containing 6 mm mixed basalt fibers. The smooth basalt fibers appear straight and needle-like, interlacing within the solid matrix. The integrity of the fiber surfaces is well-preserved, with complete fiber ends. While noticeable gaps still exist at the aggregate-matrix interface, prominent hydration products, including fibrous C-S-H and Ca(OH)_2_, are evident in the matrix. The incorporation of basalt fibers significantly enhances the matrix. The fibers provide an “anchoring” effect that improves mechanical interlocking between the fibers and the matrix. This anchoring effect, coupled with the mechanical interlocking friction during fiber extraction, collectively contributes to enhanced material properties.

## 4. Conclusions

Foam concrete specimens exhibit compressive deformation in stages: linear elastic deformation, crack initiation, crack propagation, and ultimate failure. Cracks typically extend from top to bottom and are mostly small. Basalt fibers effectively inhibit crack propagation, enhancing the specimen’s compressive strength. After splitting tensile failure, the specimen retains good integrity with an “I” shape, thanks to the bridging action of the basalt fibers.
The water–binder ratio has a great influence on the cubic compressive strength, split tensile strength, and fluidity of BF-CCG-SCFC. Silty clay reduces the cubic compressive strength, splitting tensile strength, and fluidity of BF-CCG-SCFC, while the appropriate amount of calcined gangue and basalt fiber significantly increases the cubic compressive strength and splitting tensile strength of BF-CCG-SCFC and reduces its fluidity. In order to meet the requirements of filling materials in the hollow area, the optimal water–binder ratio of BF-CCG-SCFC is 0.55, the mixing ratio of calcined gangue and pulverized clay is 2:2, and the content of basalt fiber should be 1%.
(a) A cubic compressive stress–strain curve model applicable to BF-CCG-CLLCC is investigated, revealing that the damage mode consists of five stages: compaction, linear elasticity, elastoplasticity, post-peak deformation, and stress plateau.
  (b) The modulus of elasticity and peak strain of BF-CCG-SCFC showed a general decreasing trend with the increase in water–binder ratio; the modulus of elasticity of BF-CCG-SCFC increased with the increase in calcined gangue proportion, and the corresponding increase in clay content led to the decrease in modulus of elasticity. When the calcined gangue doping was lower than 30%, the peak strain decreased with the increase in calcined gangue proportion; with the addition of basalt fiber, its value first increased and then decreased, and its value was greatly affected by the increase in fiber content. With the addition of basalt fiber, the modulus of elasticity firstly increases and then decreases, and its value is greatly affected by the fiber content, and the increase in basalt fiber doping, the peak strain shows an obvious linear growth.
The water–binder ratio affects the matrix’s strength through the non-hydration reactions of admixture particles. The inherent activity of calcined gangue and clay, along with the differences in their physical and chemical properties, leads to the occurrence of a “gradient hydration effect” during hydration. Incorporating basalt fibers into the matrix can provide an “anchoring” effect, enhancing the mechanical interlocking between the fibers and the matrix.

## Figures and Tables

**Table 1:** Basic properties of cement.

Projects	Densities /kg·m^−3^	Stability (Test Cake Method)	Initial Setting Time/min	Final Setting Time/min	Compressive Strength/MPa		Flexural Strength/MPa	
					3 d	28 d	3 d	28 d
**Index**	**/**	**/**	>60	<600	>17	>42.5	>3.5	>6.5
**Result**	3117	Qualified	176	227	30.5	43.7	6.1	8.3
**Assessment**	Qualified	Qualified	Qualified	Qualified	Qualified	Qualified	Qualified	Qualified

**Table 2:** Chemical composition of cement.

Components	SiO_2_	Fe_2_O_3_	Al_2_O_3_	CaO	MgO	TiO_2_
**Mass Fraction/%**	25.02	3.46	6.15	63.68	1.29	0.40

**Table 3:** The main chemical composition of calcined gangue.

Ingredient	SiO_2_	Al_2_O_3_	Fe_2_O_3_	CaO	MgO	TiO_2_	P_2_O_5_	K_2_O + Na_2_O	V_2_O_5_
**%**	55	27	10.52	1.25	1.73	1.32	0.13	2.14	0.91

**Table 4:** Physical properties of polymer composite cement blowing agents.

Appearance Color	Bubble Cluster Density/kg·m^−3^	Solid Content/%	PH	Dilution Factor	Foaming Power	1 h Settlement Distance/mm	1 h Urination Rate/%
Colorless And Transparent	48	168	9.2	60	21	2	22

**Table 5:** Main performance parameters of basalt fibers.

Densities/g·cm^−3^	Tensile Strength/MPa	Modulus of Elasticity/GPa	Monofilament Diameter/um	Lengths/mm	Elongation At Break/%
2.65	1256	76.1	17	6	3.21

**Table 6:** Table of influencing factors and levels in the one-way test.

Number	Water–Binder Ratio	Calcined Coal Gangue Mixing	Silty Clay Admixture	Basalt Fiber Dosage	Foaming Agent Dosage
D1	0.4	20%	20%	0.5%	5%
D2	0.45	20%	20%	0.5%	5%
D3	0.5	20%	20%	0.5%	5%
D4	0.55	20%	20%	0.5%	5%
D5	0.6	20%	20%	0.5%	5%
DGC6	0.55	0	40%	0.5%	5%
DGC7	0.55	10%	30%	0.5%	5%
DGC8	0.55	20%	20%	0.5%	5%
DGC9	0.55	30%	10%	0.5%	5%
DGC10	0.55	40%	0	0.5%	5%
DBF11	0.55	20%	20%	0%	5%
DBF12	0.55	20%	20%	0.5%	5%
DBF13	0.55	20%	20%	1.0%	5%
DBF14	0.55	20%	20%	1.5%	5%
DBF15	0.55	20%	20%	2.0%	5%

**Table 7:** Basalt fiber–calcined gangue–powdery clay foam concrete ratios (kg/m^3^).

Number	Water	Cement	Foam	Silty Clay	Calcined Gangue	Basalt Fiber
D1	220.80	331.20	27.20	110.40	110.40	4.00
D2	239.84	319.78	27.20	106.59	106.59	4.00
D3	257.60	309.12	27.20	103.04	103.04	4.00
D4	274.22	299.15	27.20	99.72	99.72	4.00
D5	289.80	289.80	27.20	96.60	96.60	4.00
DGC6	274.22	299.15	27.20	199.43	0.00	4.00
DGC7	274.22	299.15	27.20	149.58	49.86	4.00
DGC8	274.22	299.15	27.20	99.72	99.72	4.00
DGC9	274.22	299.15	27.20	49.86	149.58	4.00
DGC10	274.22	299.15	27.20	0.00	199.43	4.00
DBF11	274.22	299.15	27.20	99.72	99.72	0.00
DBF12	274.22	299.15	27.20	99.72	99.72	4.00
DBF13	274.22	299.15	27.20	99.72	99.72	8.00
DBF14	274.22	299.15	27.20	99.72	99.72	12.00
DBF15	274.22	299.15	27.20	99.72	99.72	16.00

**Table 8:** Working performance of BF-CCG-SCFC specimens and corresponding mixing ratio.

Serial Number	Water-to-Cement Ratio	m(gangue):m(clay)	Basalt Fiber Dosage	Cubic Compressive Strength/MPa	Split Tensile Strength/MPa	Fluidity/mm
Requirements of the corresponding norms				>0.6 MPa	/	160~200 mm
1	0.4	2:2	0.50%	1.76	0.82	144.8
2	0.45	2:2	0.50%	1.25	0.45	211
3	0.5	2:2	0.50%	1.45	0.65	198.5
4	0.55	2:2	0.50%	1.18	0.53	183.3
5	0.6	2:2	0.50%	0.93	0.41	295
6	0.55	0:4	0.50%	0.8	0.34	234.5
7	0.55	1:3	0.50%	0.85	0.38	223
8	0.55	2:2	0.50%	1.18	0.56	201
9	0.55	3:1	0.50%	1.57	0.77	196
10	0.55	4:0	0.50%	0.94	0.45	180.5
11	0.55	2:2	0%	1.35	0.41	235
12	0.55	2:2	0.50%	1.6	0.65	213
13	0.55	2:2	1%	1.78	0.82	200.5
14	0.55	2:2	1.50%	1.15	0.53	194.3
15	0.55	2:2	2%	1.02	0.37	190.8

**Table 9:** Correlation results.

Correlation Results of Each Index					
28d Cubic Compressive Strength		28d Split Tensile Strength		Fluidity	
Name	Correlation	Name	Correlation	Name	Correlation
Water-cement ratio	0.840	Water-cement ratio	0.831	Water-cement ratio	0.926
Basalt fiber dosage	0.774	Basalt fiber dosage	0.772	m(gangue):m(clay)	0.761
m(gangue):m(clay)	0.770	m(gangue):m(clay)	0.770	Basalt fiber dosage	0.746

## Data Availability

The original contributions presented in the study are included in the article. Further inquiries can be directed to the corresponding authors.
